# APP—A Novel Player within the Presynaptic Active Zone Proteome

**DOI:** 10.3389/fnmol.2017.00043

**Published:** 2017-02-20

**Authors:** Jens Weingarten, Melanie Weingarten, Martin Wegner, Walter Volknandt

**Affiliations:** ^1^Institute for Cell Biology and Neuroscience, Biologicum and BMLS, Goethe UniversityFrankfurt am Main, Germany; ^2^Department of Molecular Bioinformatics, Goethe UniversityFrankfurt am Main, Germany

**Keywords:** amyloid precursor protein, hippocampus, neuronal network, presynaptic active zone, synapse

## Abstract

The amyloid precursor protein (APP) was discovered in the 1980s as the precursor protein of the amyloid A4 peptide. The amyloid A4 peptide, also known as A-beta (Aβ), is the main constituent of senile plaques implicated in Alzheimer’s disease (AD). In association with the amyloid deposits, increasing impairments in learning and memory as well as the degeneration of neurons especially in the hippocampus formation are hallmarks of the pathogenesis of AD. Within the last decades much effort has been expended into understanding the pathogenesis of AD. However, little is known about the physiological role of APP within the central nervous system (CNS). Allocating APP to the proteome of the highly dynamic presynaptic active zone (PAZ) identified APP as a novel player within this neuronal communication and signaling network. The analysis of the hippocampal PAZ proteome derived from APP-mutant mice demonstrates that APP is tightly embedded in the underlying protein network. Strikingly, APP deletion accounts for major dysregulation within the PAZ proteome network. Ca^2+^-homeostasis, neurotransmitter release and mitochondrial function are affected and resemble the outcome during the pathogenesis of AD. The observed changes in protein abundance that occur in the absence of APP as well as in AD suggest that APP is a structural and functional regulator within the hippocampal PAZ proteome. Within this review article, we intend to introduce APP as an important player within the hippocampal PAZ proteome and to outline the impact of APP deletion on individual PAZ proteome subcommunities.

## Introduction

**Figure 1:**
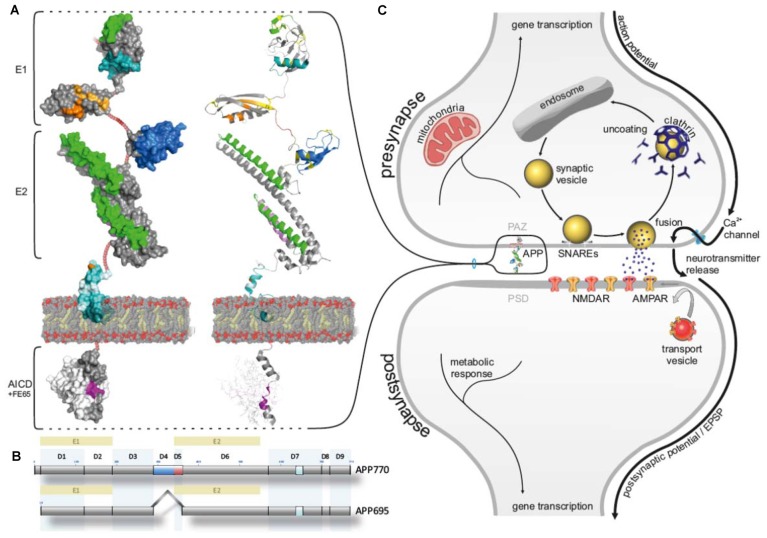
**Amyloid precursor protein (APP) allocated to the presynaptic active zone (PAZ). (A)** Schematic surface illustration (left) and cartoon (right) representing the domain organization of APP (modeling structures are created by PyMOL Molecular Graphics system based on X-ray data from protein data base, PDB; 11.2010). Heparin-binding domain/ growth factor-like domain (HBD/GFLD, green), disulfide bridges (yellow), copper-binding domain (CuBD, orange), zinc-binding domain (ZnBD, bright orange), acidic domain (DE, firebrick), Kunitz protease inhibitor domain (blue), collagene binding domain (violet), A-beta (Aβ) sequence (cyan), secretase cleavage site (pale cyan), transmembrane segment (dark teal), YENPTY sequence (magenta), NPXY sequence (purple), FE65 adaptor protein (white), non-modeled render sequences (ruby). **(B)** Subdivision of domains (D1-D9) in APP695 (expressed in neurons) and APP770 (KPI highlighted in blue, acidic domain highlighted in blue). **(C)** Schematic cartoon of a chemical synapse highlighting APP at the PAZ.

The development of a neuronal circuit requires precise coordination of billions of neurons, with up to 100,000 synaptic connections each, forming a stable but plastic network and persisting over the lifespan of an organism (Turrigiano, 2008, 2012). The key word deciphering this phenomenon is “homeostasis” and was introduced by Walter Cannon in the early 1930’s (Cannon, 1932). Within the neuronal network numerous homeostatic mechanisms ensure physiological activity in a spatio-temporal manner on various groups of synapses (Turrigiano, 2008; Yu and Goda, 2009). Maintenance of synaptic homeostasis demands on a coordinated proteomic response at both—pre- and postsynaptic sites (Schanzenbächer et al., 2016). Consecutive steps of processing the arrival of an action potential into a chemical signal by recruiting a subset of individual proteins that fuse synaptic vesicles with the presynaptic plasma membrane, release of their neurotransmitter into the synaptic cleft, which further react with their specific receptor at the postsynapse (Figure 1), demands on a rather stringent progression. Key players within this network comprise prominent candidates like synaptic vesicle protein 2 (SV2), synaptotagmin-1, synaptosomal associated protein 25 (SNAP25), syntaxin and vesicle associated membrane protein2/synaptobrevin2 (VAMP2; Südhof and Rizo, 2011; Südhof, 2012; Laßek et al., 2015). The unique set of proteins regulating, mediating and controlling proper presynaptic physiology was recently complemented by a yet unappreciated companion—the amyloid precursor protein (APP).

## App—A Brief Profile

The APP belongs to an evolutionary conserved gene family with specific expression pattern in *C. elegans, Drosophila* and mammals (reviewed in Coulson et al., 2000; Jacobsen and Iverfeldt, 2009). Discovered during the 1980s as precursor protein of A-beta (Aβ)—the main constituent of senile plaques—much effort has been made to understand the pathophysiology of Alzheimer’s disease (AD) and the physiological function of APP (Glenner and Wong, 1984; Kang et al., 1987). The progression of AD is characterized by a massive loss of synapses especially within the hippocampus. Extracellular senile plaques and intracellular neurofibrillary tangles induce and promote successive degeneration of neurons manifested by severe impairments in learning and memory and behavioral changes (Grundke-Iqbal et al., 1986; Supnet and Bezprozvanny, 2010). Enzymatic processing of APP is initiated by either β-secretase/γ-secretase (amyloidogenic pathway) cleavage, or α-secretase/γ- secretase (non-amyloidogenic pathway). The proteolytic processing of amyloid precursor like proteins 1 and 2 (APLP1 and APLP2) is comparable to that of APP, however, only the amyloidogenic pathway can induce the formation of Aβ-peptides (Eggert et al., 2004). Until now, little is known about the shift in enzymatic processing of the APP leading to the accumulation of Aβ-peptides and the formation of oligomers and fibrils. Since senile plaques consist mainly of Aβ-fibrils, it was of great interest how these structures are organized (Lu et al., 2013). The analysis of these fibrils derived from AD patients revealed an individual molecular structure. These variations were suggested to correlate with the severity of impairments in the individual pathogenesis of AD in patients (Lu et al., 2013).

APP is a type 1 transmembrane protein with a large N-terminal domain, a single transmembrane region and a short C-terminal domain (Figure 1). The N-terminal domain is subdivided into an E1 domain comprising a heparin-binding/growth factor-like domain (HBD/GFLD), a copper and zinc-binding domain (CuBD/ZnBD) followed by an acidic region (DE), optionally a KPI-domain (not present in the neuronal specific isoform APP695), and an E2 domain consisting of a second HBD (HBD2) a collagen-binding region and N-glycosylation binding sites (Jacobsen and Iverfeldt, 2009; Kaden et al., 2012). The APP intracellular domain (AICD) contains the highly conserved YENPTY motif involved in the internalization of APP and phosphorylated or dephosphorylated tyrosine mediated binding of adaptor proteins like FE65 (Figure 1), Dab1 and X11a (munc-18 interacting protein, Mint; Jacobsen and Iverfeldt, 2009). All APP family members reveal a high structural overlap except the Aβ domain that is only present in mammalian APP.

## App at the Synapse

Multiple isoforms were described for mammalian APP (e.g., 695aa, 770aa), but only APP695aa is expressed in neurons. Within neurons, APP was discussed as* bona fide* SV (Groemer et al., 2011) and constituent of the presynaptic plasma membrane (Marquez-Sterling et al., 1997; Lyckman et al., 1998). In addition, APP was described as a constituent of endocytosed synaptic vesicles, but being sorted away from* bona fide* synaptic vesicles (Marquez-Sterling et al., 1997). On the contrary, Groemer et al. (2011) reported a small copy number of APP to synaptic vesicles as a result of endosomal synaptic vesicle recycling processes. However, they emphasized that the majority of APP was immunodetected in fractions containing the plasma membrane, and only a small amount was present in purified synaptic vesicle fractions (Groemer et al., 2011). In our studies, we clearly demonstrated that APP and its family members are constituents of the presynaptic plasma membrane and that APP is virtually absent from synaptic vesicles (Laßek et al., 2013). Within the presynaptic nerve terminal, a small section characterized by an assembly of electron dense material, is responsible for Ca^2+^-triggered exocytosis of synaptic vesicles. This section is called presynaptic active zone (PAZ; Gray, 1963; Südhof, 2012). The composition of the PAZ proteome identified the release site as dynamic focal hot spot, providing the prerequisite for structural and functional changes also in the adult nerve terminal. Neuronal communication and signal transduction depends not only on the concerted action of individual proteins within the PAZ but also on proper energy supply (Boveris and Navarro, 2008). Besides the glycolytic chain associated with synaptic vesicles (reviewed in Burré and Volknandt, 2007), mitochondria are the main source for the production of ATP at the presynaptic terminal. Therefore, mitochondria are essential in maintaining presynaptic homeostasis and phosphorylation reactions and are highly involved in synaptic plasticity (reviewed in Mattson et al., 2008). The allocation of APP to the proteome of this highly dynamic substructure of the presynapse, identified APP as yet unknown player within the neuronal communication and signaling network (Laßek et al., 2013, 2016).

To address the question which physiological function APP is executing in the central nervous system (CNS), a variety of genetically designed mouse models has been generated (Heber et al., 2000; Ring et al., 2007; Hick et al., 2015). It turned out that loss of APP causes an age-dependent phenotype with no severe physiological impairments in younger mice but impairments in learning and memory in the elderly (Phinney et al., 1999; Ring et al., 2007). At postsynaptic sites, reduced dendritic length and branching accompanied by a total spine density reduction was characteristic for old APP-KO mice and indicates a physiological role of APP in maintaining spine density (Tyan et al., 2012). This was further supported by Weyer et al. (2014) demonstrating a specific role of APP in sustaining spine structure and density. Classification of spine structure can be morphologically addressed revealing stubby, thin and mushroom spines. In APP-KO cornu ammonis 1 (CA1) neurons this spine subtype distribution is altered by a significant decrease in mushroom spines (Weyer et al., 2014). Interestingly, substantial changes of the proteomic composition of neurotransmitter release sites are already detectable in younger mice (Laßek et al., 2014, 2016). Since APP plays an essential role during the development of the neuronal circuit (Lazarov and Demars, 2012), it was suggested that the APLP2 compensates for the loss of APP (Weyer et al., 2011; Laßek et al., 2016). Screening immunopurified PAZs derived from individual total mouse brain revealed prominent players to be affected by APP deletion. Candidates like SV2A, synaptotagmin-1 and synaptophysin turned out to be differentially regulated. It is worth mentioning, that the opposite effect was observed for deletion of either APLP1 or APLP2 (Laßek et al., 2014). Moreover, deletion of the family members did not result in any morphological alterations in CNS or overall impairments in learning and memory (Heber et al., 2000; Weyer et al., 2011). Memory formation requires a variety of network oscillations that are regularly synchronized between hippocampal CA1 and CA3 region (Korte et al., 2012). As inhibitory interneurons play an essential role in this coordinated action of synchronization, their oscillations can affect a large population of pyramidal neurons, inhibiting specific input pathways and guarantee for a high background-to-noise ratio (Mann and Paulsen, 2007). Accompanied with the observed shift in the excitatory-inhibitory ratio in APP-KO mice, it was suggested that GABAA receptor-mediated inhibition is altered in aged APP-KO mice and that these changes contribute to the reduction in LTP in aged APP-KO mice. This assumption was further sustained by LTP-rescue experiments employing pharmacological blockade of GABA_A_ receptors (Fitzjohn et al., 2000; Korte et al., 2012). Synaptic plasticity requires persistent changes within the entire network. Thereby, the strength of a neuronal connection is individually adjusted (up or down) dependent on homeostatic synaptic scaling (Turrigiano, 2012; Davis, 2013). Homeostasis implies the capability to restore individual baseline functions upon continued input. This is achieved by controlling and modulating the expression and trafficking of specific proteins and protein complexes. Initially, synaptic scaling was described as bidirectional modulation of neurotransmitter receptor abundance at individual synapses. In this context, it was suggested that this effect stabilizes neuronal excitability while sustaining learning-related information (Turrigiano, 2008, 2012; Davis, 2013). At presynaptic sites, homeostasis encompasses the fast, long-lasting and accurate modulation of synaptic vesicle fusion (Davis, 2013). Alteration at protein level as response to homeostatic scaling in hippocampal neurons was recently analyzed by Schanzenbächer et al. (2016). They uncovered the necessity of new protein synthesis upon up- or down scaling induced by pharmacological treatment. More than 300 proteins (e.g., neurotransmitter receptors, scaffolding and signaling proteins) were affected by this stimulation. Strikingly, genes affected by the stimulation, encode for proteins critically involved in neurological diseases like AD, Parkinson’s disease (PD) or schizophrenia. Proteins identified and regulated by homeostatic scaling in this approach provide a starting point to examine how their dysregulation might contributes to a variety of neuronal disorders (Schanzenbächer et al., 2016).

## App and the Hippocampus

APP is functionally integrated into the hippocampal PAZ proteome and fits into the evolutionary conserved active zone protein complex, comprising prominent constituents like ELKS, CASK bassoon, RIM and Munc18 (Südhof, 2012; Laßek et al., 2016). Embedding APP into the entire PAZ proteome unraveled APP as a context-sensitive regulator with impact on synaptic vesicle cycle, cytoskeletal organization and Ca^2+^-homeostasis. Deletion of APP significantly affects those proteins serving as mediator (e.g., α-synuclein) within the PAZ but not their central players (e.g., SNARE-machinery). It was obvious, that loss of APP accounts for individual rearrangements of the entire network structure with no current effect on presynaptic functionality (Laßek et al., 2016). Interestingly, these massive alterations in protein abundance within the PAZ proteome did not account for impairments in learning and memory pointing to a yet unknown compensatory mechanism in young APP-KO mice (Ring et al., 2007). The most important guarantors for sufficient energy metabolism, calcium- and redox homeostasis are mitochondria (Yin et al., 2014; Grimm et al., 2016). They support the intracellular energy demand by producing ATP, affect redox-sensitive kinases via second messengers H_2_O_2_ and NO and regulate the NAD+/NADH homeostasis, involved in maintenance of mitochondrial energy statues (Yin et al., 2014). A recent proteome study on young and old APP-KO mice revealed drastic changes in mitochondrial protein abundance at hippocampal neurotransmitter release sites. These results indicated that old APP-KO mice display a dysregulation in their bioenergetics metabolism accompanied by hyperphosphorylation of CaMKII (Laßek et al., 2017). It is tempting to speculate that during the induction of LTP CaMKII becomes over-activated, which has a negative impact on synaptic plasticity, and prevents proper learning and memory consolidation. Recently, over-activation of CaMKII was described in hippocampal neurons following synaptic stimulation and increased intracellular Ca^2+^-levels. As a kind of protective mechanism, CaMKII is able to form clusters (spherical clusters, identical in size and shape) preventing excessive protein phosphorylation, independent of the autocatalytic center, due to an imbalance in Ca^2+^-homeostasis (Dosemeci et al., 2007). Therefore, cognitive impairments in old APP-KO mice might be associated with an imbalance in mitochondrial function and phosphorylation-activity of the serine/threonine-specific kinases CaMKII as observed during the progression of AD (Grimm et al., 2016). Thermodynamic imbalance and compensatory mechanisms acting in impaired neurons, will further induce a competition for energy substrates and finally shift formerly healthy neurons into affected ones (Demetrius et al., 2015).

The expression pattern of APP in the hippocampus and especially at PAZs has been further analyzed in detail by Rodrigues et al. (2014), demonstrating that APP is most abundant on glutamatergic neurotransmitter release sites as compared to GABAergic ones. Their findings further revealed that less than half of hippocampal synapses were immunopositive for APP (Rodrigues et al., 2014). Strikingly, deletion of APP accounts for an increase in activity of GABAergic synapses. This dysregulation in balance between inhibitory and excitatory neurons was induced by a diminished endocytosis of VDCC in GABAergic hippocampal neurons (Yang et al., 2009). In this context it is worth mentioning the idea of graded molecular profiles of hippocampal neurons (here CA1 neurons) stated by Cembrowski et al. (2016). The hippocampal formation has been attributed with regional-specific functions. Whereas the dorsal hippocampus is known to be associated with cognitive functions (like memory and spatial navigation) the ventral region is basically associated with behavior (like stress and emotion; Fanselow and Dong, 2010; Strange et al., 2014). Illustrating the gene expression profile along the dorsal-ventral-axis of CA1 neurons revealed unique profiles of decay. These findings make it rather interesting to figure out how those CA1 neurons perform their region-specific functions. In line with these findings similar considerations were made for the diversity of presynaptic performance. Atwood and Karunanithi (2002) described various models of functional differentiation of presynaptic neurons. (1) Different amounts of strength-regulating presynaptic proteins or a variable combination of more than one presynaptic protein can be induced or attracted by a postsynaptic neuron. (2) Neuronal activity or specific input to presynaptic neurons can induce a differential occurrence of presynaptic proteins in different neurons (Atwood and Karunanithi, 2002). However, if the molecular profile and physiology of those neurons is different, what about the profile of individually expressed proteins? The abundance of several PAZ proteins differs considerably between brain regions, presumably reflecting region-specific functional adaptions. This can be of vital importance to understand the impact of therapeutic drugs (e.g., prevalence or therapy of AD) on their targets and to elucidate their subsequent effects on the PAZ proteome. The identification of individual PAZ protein components is a prerequisite for further functional investigations and also provides a solid basis for evaluating their interaction. Therefore, differences in the PAZ proteome reflect specific adaptions to regional neuronal circuitries and the functional and structural dynamics of their corresponding release sites (Weingarten et al., 2015). Salient findings by Schwenk et al. (2014) are in accordance with this dynamic and functional diversity of proteomes. They demonstrated that a large multiprotein complex provides an individual assembly of its core-subunits and a regional specific architecture over space and time (Schwenk et al., 2014). This perspective is indispensable for those proteins sharing a differential expression pattern (like APP)—not only in specific brain regions but also in neurons and individual synapses, respectively. A current study by Counts et al. (2014) went a step further, performing molecular profiling of CA1 neurons derived from patients with mild cognitive impairment (MCI) and AD—MCI, is a prodromal stage of AD. It is widely accepted that early pathological events triggering the outcome of AD are associated with CA1 neurons. Compared to control groups (NCI, no cognitive impairments), expression of genes involved in proper synaptic function in CA1 neurons, is severely dysregulation in MCI, whereas no further changes were observed in AD. Interestingly, APP, APLP1 and APLP2 transcripts were not altered at any stage in CA1 neurons. Molecular profiling of CA1 neurons revealed that early changes in synaptic elements provide susceptibility to cognitive decline in aged patients. These findings point to an early onset of synaptic failure that becomes manifested in the dysregulation of the hippocampal neuronal circuit (Counts et al., 2014).

## Concluding Remarks

Our proteomic profiling of PAZs derived from total mouse brain revealed a summary of all alterations due to loss of APP. Going a step further, looking only at the hippocampus, the picture of presynaptic changes was impressively refined. Therefore, the next step should include proteomic studies addressing the molecular profiling of individual neurons within the hippocampus. Combining our approaches with new technologies will provide novel insights into the biological function of APP within the CNS. Moreover, interdisciplinary approaches and sustained exchanges of information can facilitate new perspectives within the challenging APP research field.

## Author Contributions

The authors MWei, JW, MWeg and WV contributed equally to this review article.

## Conflict of Interest Statement

The authors declare that the research was conducted in the absence of any commercial or financial relationships that could be construed as a potential conflict of interest. The handling Editor declared a shared affiliation, though no other collaboration, with the authors JW, MWei, MWeg and WV, and the handling Editor states that the process met the standards of a fair and objective review.

## Abbreviations

AD: Alzheimer’s disease
APLP1/2: amyloid precursor like proteins1 and 2
APP: amyloid precursor protein
CA: cornu ammonis
HD: Huntington’s disease
PAZ: presynaptic active zone
PD: Parkinson’s disease
SNAP25: synaptosomal associated protein 25
SV2: synaptic vesicle protein 2
VAMP2: vesicle associated membrane protein2/synaptobrevin2.
